# *Tuber* Inoculation Drives Rhizosphere Microbiome Assembly and Metabolic Reprogramming in *Corylus*

**DOI:** 10.3390/ijms27020768

**Published:** 2026-01-12

**Authors:** Jing Wang, Nian-Kai Zeng, Xueyan Zhang

**Affiliations:** 1 Ministry of Education Key Laboratory for Ecology of Tropical Islands, Key Laboratory of Tropical Animal and Plant Ecology of Hainan Province, College of Life Sciences, Hainan Normal University, Haikou 570228, China; wj630203782@163.com; 2 Guizhou Institute of Biology, Guizhou Academy of Sciences, 1 Longjiang Lane, Guiyang 550009, China

**Keywords:** rhizosphere microbiome, metabolic reprogramming, multi-omics integration, community assembly, functional succession

## Abstract

To elucidate the potential of integrated multi-omics approaches for studying systemic mechanisms of mycorrhizal fungi in mediating plant-microbe interactions, this study employed the *Tuber*-inoculated *Corylus* system as a model to demonstrate how high-throughput profiling can investigate how fungal inoculation reshapes the rhizosphere microbial community and correlates with host metabolism. A pot experiment was conducted comparing inoculated (CTG) and non-inoculated (CK) plants, followed by integrated multi-omics analysis involving high-throughput sequencing (16S/ITS), functional prediction (PICRUSt2/FUNGuild), and metabolomics (UPLC-MS/MS). The results demonstrated that inoculation significantly restructured the fungal community, establishing *Tuber* as a dominant symbiotic guild and effectively suppressing pathogenic fungi. Although bacterial alpha diversity remained stable, the functional profile shifted markedly toward symbiotic support, including antibiotic biosynthesis and environmental adaptation. Concurrently, root metabolic reprogramming occurred, characterized by upregulation of strigolactones and downregulation of gibberellin A5, suggesting a potential “symbiosis-priority” strategy wherein carbon allocation shifted from structural growth to energy storage, and plant defense transitioned from broad-spectrum resistance to targeted regulation. Multi-omics correlation analysis further revealed notable associations between microbial communities and root metabolites, proposing a model in which *Tuber* acts as a core regulator that collaborates with the host to assemble a complementary micro-ecosystem. In summary, the integrated approach successfully captured multi-level changes, suggesting that *Tuber*-*Corylus* symbiosis constitutes a fungus-driven process that transforms the rhizosphere from a competitive state into a mutualistic state, thereby illustrating the role of mycorrhizal fungi as “ecosystem engineers” and providing a methodological framework for green agriculture research.

## 1. Introduction

Mycorrhizal symbiosis is one of the most crucial mutualistic relationships between plants and fungi in nature. Among these, ectomycorrhizal (ECM) fungi play an indispensable role in nutrient acquisition for forest trees and overall ecosystem functioning [1]. *Tuber*, a valuable genus of ECM fungi, has garnered significant research interest due to its ability to form complex metabolic interactions with host plants and perform key ecological regulatory functions [2,3]. Recent phylogenetic and genomic analyses have further illuminated the evolutionary trajectory of the Tuberaceae family, highlighting the genetic toolkit that underpins their unique symbiotic lifestyle and secondary metabolite production, which are critical for their ecological success [4,5]. Studies have shown that *Tuber* colonization enhances the host plant’s (e.g., *Corylus*, *Quercus*, *Pinus*) uptake of mineral elements such as nitrogen and phosphorus, while improving resistance to biotic and abiotic stresses [6,7,8]. *Corylus* is an important economic tree species, yet intensive cultivation often leads to challenges like soil fertility decline and accumulation of soil-borne pathogens. Therefore, using mycorrhizal technology, particularly *Tuber* inoculation, to optimize the rhizosphere microenvironment has become a key strategy for sustainable hazelnut production [9,10]. Although *Tuber* inoculation is known to promote hazelnut growth [8,9,10,11], the mechanisms through which this symbiosis systemically reshapes the structure and function of the rhizosphere microbial community and coordinates the host’s metabolic responses remain poorly understood, particularly regarding how *Tuber* acts as a core regulator to integrate multi-kingdom interactions and drive the micro-ecosystem toward a functional synergy [12].

Mycorrhizal fungi are regarded as “ecosystem engineers” in the rhizosphere. Through extensive mycelial networks and specific metabolic secretions (e.g., extracellular enzymes, organic acids, antimicrobial compounds) [13], they modify the physical structure, chemical environment, and biological composition of the rhizosphere [14,15]. For example, *Tuber* can suppress soil-borne pathogens such as *Fusarium* by producing antimicrobial volatiles [16,17], and improve host plant nutrition by secreting organic acids that solubilize insoluble phosphorus [2]. These fungal-driven changes significantly influence microbial community assembly. Research shows that mycorrhizal symbiosis promotes functional succession within the rhizosphere microbiome-shifting from r-strategist bacteria (e.g., *Bacteroidota*), which thrive on simple root exudates, toward K-strategist bacteria (e.g., *Actinobacteriota*), which decompose complex organic matter [18]. Emerging approaches, such as metatranscriptomics and stable isotope probing, have enabled real-time tracking of nutrient fluxes and gene expression in mycorrhizal systems, providing deeper insights into microbial functional dynamics [19,20,21]. Concurrently, the host plant undergoes metabolic reprogramming to establish and maintain symbiosis, including adjustments to hormonal signaling (e.g., upregulation of strigolactones, downregulation of gibberellins), reallocation of carbon (from structural growth to energy storage), and a shift in defense strategy (from basal to precision regulation) [22,23]. This reprogramming often reflects a trade-off between growth and defense, a concept supported by recent metabolomic work on mycorrhizal plants [24]. Thus, successful mycorrhizal symbiosis represents a functionally optimized micro-ecosystem co-constructed through chemical dialogue among the plant, the fungus, and the rhizosphere microbiota.

Recent advances in multi-omics technologies provide powerful tools for deciphering these complex interactions [25]. Integrating microbiome analysis (e.g., high-throughput sequencing of 16S and ITS) with metabolomics (e.g., non-targeted UPLC-MS/MS) allows simultaneous assessment of community structure and metabolic function [26], revealing systemic changes induced by mycorrhizal symbiosis [27,28]. Emerging approaches, such as metatranscriptomics and stable isotope probing, have enabled real-time tracking of nutrient fluxes and gene expression in mycorrhizal systems, providing deeper insights into microbial functional dynamics [19]. Statistical approaches such as Procrustes analysis can quantify correlations between microbial community structure and metabolic profiles [29], while correlation networks help identify interactions between key microbial taxa and metabolites [30]. However, a critical knowledge gap persists in understanding how ECM fungi, particularly *Tuber*, coordinate the assembly of rhizosphere microbiomes and host metabolic networks to transition the micro-ecosystem from a diverse but competitive state to a streamlined, symbiotic state [31,32]. Conventional theory posits that plants prioritize “autonomous growth” in the absence of symbiotic partners, allocating resources predominantly to their own reproduction and survival [33]. Upon the establishment of mycorrhizal symbiosis, however, plants undergo metabolic reprogramming to support the growth and functionality of fungal partners, shifting toward a “symbiosis-priority” strategy [4]. This transition reflects a fundamental adaptation in plant resource allocation, yet the molecular switches and precise regulatory networks governing this shift remain incompletely elucidated [34].

To address this, we used *Corylus heterophylla* × *C. avellana* inoculated with *Tuber* as a model system. Using integrated microbiome–metabolome analysis, we aimed to elucidate three core questions: (1) What are the effects of *Tuber* inoculation on the structural diversity, composition, and functional potential of bacterial and fungal communities in the hazelnut rhizosphere? (2) What key metabolic changes occur in hazelnut roots during *Tuber* symbiosis, and how do they contribute to the establishment and maintenance of the symbiotic state? (3) How do correlations between rhizosphere microbiome shifts and root metabolic reprogramming reflect the integrative role of *Tuber* in synchronizing plant-microbial responses? This study seeks to uncover the micro-ecological mechanisms of *Tuber*-*Corylus* symbiosis from a systems perspective, providing a theoretical basis for directed manipulation of soil microecology and sustainable agriculture through beneficial mycorrhizal fungi.

## 2. Results

### 2.1. Soil Microbial Community Structure and Functional Insights

#### 2.1.1. Soil Microbial Diversity Analysis

**Figure 1:**
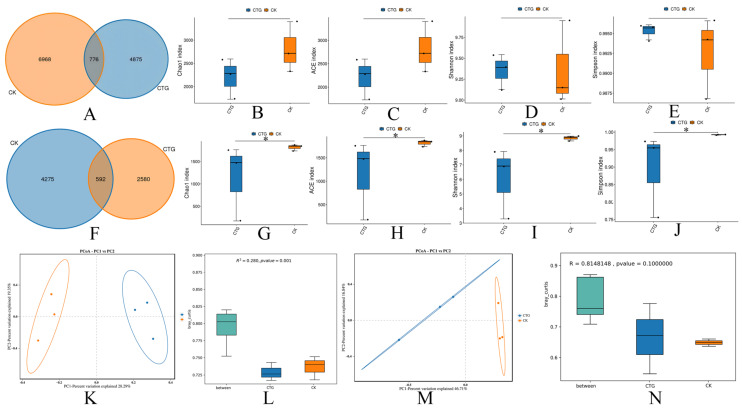
Comparative analysis of soil microbial diversity between non-inoculated control (CK) and *Tuber*-inoculated (CTG) treatments. (**A**,**F**) Venn diagram; (**B**,**G**) Chao 1 index; (**C**,**H**) ACE index; (**D**,**I**) Simpson index; (**E**,**J**) Shannon index; (**K**,**M**) PCoA; (**L**,**N**) anosim index; (**A**–**E**,**K**,**L**) bacteria; (**F**–**L**,**M**,**N**) fungi; * indicates *p* < 0.05.

As summarized in Figure 1, *Tuber* exerted divergent effects on bacterial and fungal communities in the hazelnut rhizosphere. For bacteria, differences in the number of species detected were observed between the non-inoculated control (CK) and the *Tuber*-inoculated group (CTG) (Figure 1A). Analysis of alpha diversity indices (Chao1, ACE, Shannon, and Simpson) (Figure 1B–E), coupled with beta diversity assessments based on PCoA (Bray-Curtis distance) (Figure 1K) and ANOSIM (Figure 1L), revealed discernible but statistically non-significant differences in both within-sample (alpha) and between-sample (beta) diversity between the non-inoculated control (CK) and the *Tuber*-inoculated (CTG) groups. This suggests that *Tuber* inoculation did not substantially affect the richness or evenness of the rhizobacterial community, indicating either structural resilience or functional redundancy among bacteria in response to mycorrhizal colonization. In contrast, *Tuber* inoculation markedly influenced the fungal community. The Venn diagram (Figure 1F) clearly illustrates the distribution of fungal taxa unique to and shared between the non-inoculated control (CK) and *Tuber*-inoculated (CTG) groups, revealing a substantially higher number of unique taxa in CK, a lower number in CTG, and a relatively small shared taxa count-collectively indicating that inoculation not merely reduced species richness but fundamentally restructured the microbial community. This finding is supported by significant declines in alpha-diversity indices: Chao1 (Figure 1G) and ACE (Figure 1H), alongside reduced Shannon and Simpson indices (Figure 1I,J), which together suggest a simplification of community structure likely due to the dominance of a few taxa, such as *Tuber*. Clear separation between CTG and CK samples was visually evident in the PCoA plot (Figure 1M), particularly along the first principal coordinate (PC1, explaining 46.71% of variation), highlighting the treatment effect. Although the ANOSIM result (Figure 1N) did not reach statistical significance (*p* > 0.05), the directional trend indicated by its R-value, consistent with the uniform reductions across all major alpha-diversity indices and the clear separation in PCoA space, collectively provides evidence for a treatment effect on the fungal community. In summary, alpha- and beta-diversity analyses demonstrate that *Tuber* inoculation significantly reshaped the hazel root fungal community, reducing species richness and diversity, and promoting a distinct, symbiosis-oriented assemblage centered on the *Tuber*–*Corylus* partnership.

Employing a single-time-point design, with sampling conducted six months after inoculation when the symbiotic state had stabilized, this study demonstrated that *Tuber* inoculation exerted a community-specific effect: the bacterial assemblage remained largely unchanged, whereas the fungal community in the inoculated group (CTG) shifted toward a symbiosis-oriented assemblage with reduced diversity. This restructured state, marked by mycorrhizal dominance, is interpreted as a form of functional optimization rather than ecological dysfunction. Through enhanced nutrient acquisition and pathogen suppression [35,36], it reflects a transition in the rhizosphere from high diversity to high functionality—a shift that may contribute to improved crop health and yield in sustainable agricultural systems.

#### 2.1.2. Dominant Microbial Population Shifts

**Figure 2:**
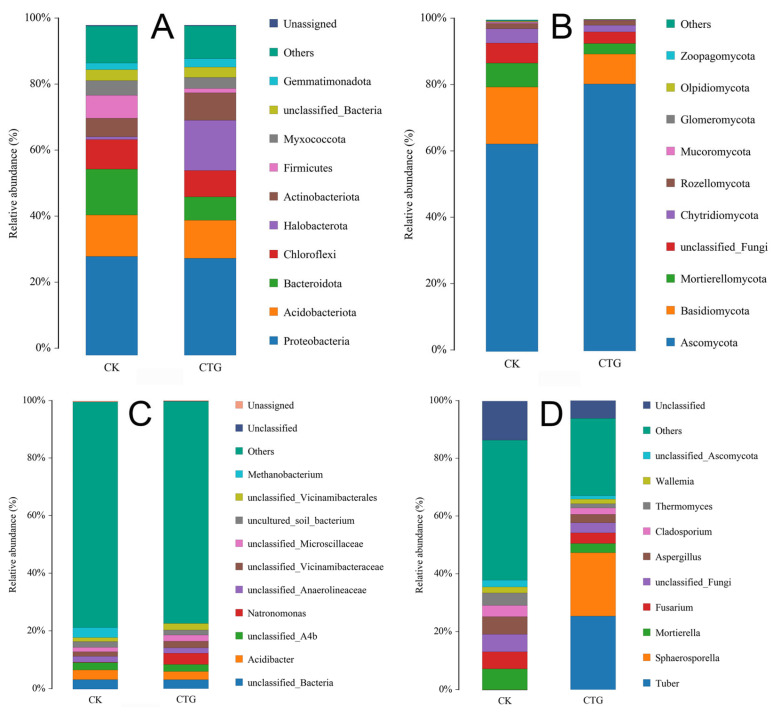
Relative abundance of soil bacterial (**A**,**C**) and fungal (**B**,**D**) communities in control (CK) and *Tuber*-inoculated (CTG) treatments at the phylum and genus levels.

At the phylum level, the bacterial community was dominated by Proteobacteria (22.72–38.79%), Acidobacteriota (8.41–19.81%), Bacteroidota (5.19–17.84%), and Chloroflexi (6.21–9.92%)—typical core taxa of soil bacterial communities [18,37]. Compared to the non-inoculated control (CK), the *Tuber*-inoculated treatment (CTG) significantly increased the relative abundances of Actinobacteriota and Halobacterota, while markedly reducing those of Bacteroidota and Firmicutes (Figure 2A). Actinobacteriota are considered K-strategists with strong abilities to decompose organic matter, mineralize nutrients, and produce antibiotics [38]. Their increase serves as a biomarker of improved rhizosphere health under mycorrhizal symbiosis [39]. Halobacterota may enhance mycorrhizal colonization by secreting extracellular polysaccharides and improving soil structure [40], while their halotolerance supports symbiosis stability under salinity stress [41,42]. In contrast, Bacteroidota and Firmicutes are typically r-strategists [18]. Their decline reflects a *Tuber*-induced shift in carbon flow and micro-environment, likely redirecting nutrient cycling from rapid mineralization of simple exudates toward mycelia-mediated decomposition of complex organics. This transition from r- to K-strategists indicates a more stable and efficient bacterial community supported by extensive mycelial networks. The stability of core phyla such as Acidobacteriota and Proteobacteria suggests that mycorrhization fine-tuned—rather than disrupted—the bacterial community structure, consistent with the unchanged alpha-diversity. In the fungal community (Figure 2B), the non-inoculated control (CK) was co-dominated by Ascomycota and Basidiomycota, typical of healthy soils [43,44]. In contrast, CTG fundamentally altered this structure: Ascomycota increased dramatically and became dominant, while Basidiomycota declined significantly. This shift resulted directly from the inoculated ectomycorrhizal fungus, *Tuber*, which belongs to Ascomycota. Its explosive abundance confirms successful colonization and establishment [45], indicating taxon-specific promotion during symbiosis.

At the genus level, the bacterial profile (Figure 2C; Table S1) showed high proportions of unclassified taxa (“Others”: CK 78.41%, CTG 77.17%), reflecting the rhizosphere’s high microbial diversity. CTG strongly reduced *Methanobacterium* abundance (from 3.53% to 0.004%), indicating improved soil aeration due to mycorrhization [46], which shifted archaeal communities toward aero-tolerant lineages [47]. Conversely, *Natronomonas* increased in CTG (3.86% vs. 0.095% in CK), suggesting mycorrhiza-induced pH neutralization or ion changes that favored its niche. Thus, *Tuber* inoculation selectively modified the rhizosphere habitat—enhancing aeration and altering chemistry—to suppress anaerobic taxa while promoting specialists adapted to new conditions. The fungal genus profile (Figure 2D; Table S2) revealed substantial enrichment of *Tuber* and *Sphaerosporella* in CTG. High *Tuber* abundance confirmed successful inoculation and dominance [45]. *Sphaerosporella*—another ectomycorrhizal fungus in the order Pezizales—was co-enriched, likely due to a *Tuber*-induced “fungal-friendly” environment rich in carbon exudates and signaling molecules [48]. This promoted spore germination and colonization of compatible mycorrhizal fungi from the soil or air [49], forming a composite symbiotic community with *Tuber* as the core pioneer, integrated with native beneficial fungi [50]. Together, they constructed a healthier and more efficient rhizosphere micro-environment for the host plant [1].

#### 2.1.3. Intergroup Differential Analysis

**Figure 3:**
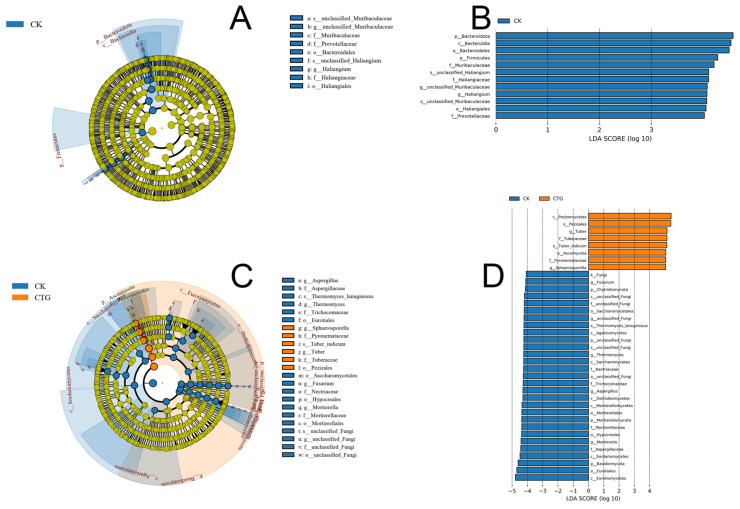
Differential soil microbial taxa between control (CK) and *Tuber*-inoculated (CTG) groups identified by linear discriminant analysis effect size (LEfSe). (**A**,**B**) Bacterial taxa; (**C**,**D**) fungal taxa.

LEfSe analysis (LDA > 4.0) identified statistically significant biomarkers between the CK and CTG rhizosphere microbial communities [51]. For bacteria (Figure 3A,B), the CK group exhibited enrichment of six dominant taxa: Bacteroidota, Bacteroidia, Bacteroidales, Firmicutes, Muribaculaceae, and lineages related to *Haliangium* (including unclassified *Haliangium*, Haliangiaceae, and Haliangiales), along with Prevotellaceae (*p* ≤ 0.01). In contrast, no bacterial taxa were significantly enriched in the CTG group. This result, combined with the enrichment of r-strategist bacteria in CK, indicated that the inoculation treatment exerted an indirect and holistic influence on the bacterial community. This influence was likely mediated by alterations in rhizosphere carbon sources, driving a functional succession from r-strategists, which preferentially utilize simple carbon sources, toward K-strategists, which are more adept at decomposing complex organic matter.

Fungal communities showed stark treatment-specific divergence (Figure 3C,D). The CTG soils were dominated by mycorrhiza-associated taxa: Ascomycota, Pezizomycetes, Pezizales, *Tuber* (including *T. indicum*), Tuberaceae, Pyronemataceae, and *Sphaerosporella* (*p* < 0.001). Conversely, the CK soils harbored higher abundances of non-symbiotic fungi, including *Fusarium*, Chytridiomycota, Saccharomycetales, *Thermomyces lanuginosus*, Agaricomycetes, and *Aspergillus*, along with Mortierellomycota lineages (Mortierellales, Mortierellaceae, *Mortierella*) and Eurotiomycetes (*p* ≤ 0.05). LEfSe results demonstrated a highly significant divergence in fungal community composition between treatments. This divergence followed a clear functional pattern: CTG enriched a complete community of symbiotic mycorrhizal fungi, while CK maintained a diverse community dominated by saprotrophic and potentially pathogenic non-symbiotic fungi.

The biomarkers in CTG soil traced a coherent taxonomic pathway from phylum Ascomycota to the class Pezizomycetes, the order Pezizales, and ultimately to the families Tuberaceae and Pyronemataceae. The analysis precisely identified the genus *Tuber*, including the species *T. indicum*, providing compelling evidence for successful colonization and dominance of the inoculated mycorrhizal fungus. The co-enrichment of Pyronemataceae and its genus *Sphaerosporella*, also an ectomycorrhizal fungus, was a key finding. This suggested that mycorrhizal inoculation not only introduced the target species but also enriched a beneficial “ectomycorrhizal fungal functional guild.” The coexistence of *Sphaerosporella* with *Tuber* implied potential niche differentiation (e.g., in nutrient acquisition or micro-environmental preferences), reducing direct competition and achieving functional complementarity to jointly promote plant health.

In contrast, the fungal community in CK soil was dominated by non-symbiotic types, including several potentially harmful taxa. The high abundance of *Fusarium*, a notorious plant pathogen, indicated a higher disease risk in CK. The genus Aspergillus also included species potentially harmful to plants. Taxa such as Chytridiomycota, Mortierellomycota, and Agaricomycetes are typically saprotrophic and primarily involved in organic matter decomposition. This indicated that the nutritional mode of the CK community was fundamentally different from that of CTG, existing in a “basal” state dominated by decomposition. The significant reduction in pathogenic (e.g., *Fusarium*) and saprotrophic (e.g., *Mortierella*) fungi in CTG was a classic manifestation of the competitive exclusion principle [52]. This suggested that symbiotic fungi like *Tuber* and *Sphaerosporella* effectively preempted root infection sites and, as strong carbon sinks, consumed host photosynthates preferentially, likely depriving pathogens and saprotrophs of essential space and nutrients and resulting in effective biocontrol.

#### 2.1.4. Functional Gene Prediction

**Figure 4:**
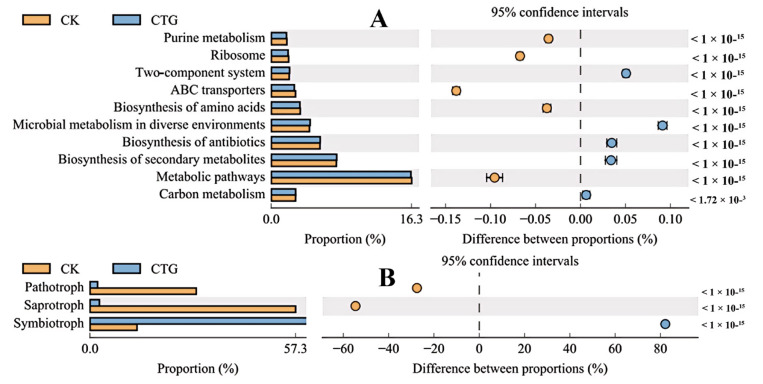
KEGG pathway enrichment analysis in control (CK) and *Tuber*-inoculated (CTG) treatments. (**A**) Bacterial community; (**B**) fungal community.

Functional prediction of bacteria using PICRUSt2 and KEGG pathway enrichment analysis (Figure 4A) revealed that truffle mycorrhization (CTG) induced a significant shift in the functional potential of the rhizobacterial community. This shift reflected a transition from basic metabolic maintenance toward adaptation to the symbiotic environment and active participation in symbiosis establishment. Pathways including purine metabolism, ribosome function, ABC transporters, amino acid biosynthesis, and general metabolic pathways were enriched in the non-mycorrhizal control (CK), indicating that bacteria in the absence of mycorrhiza allocated substantial resources to genetic replication, protein synthesis, and competitive nutrient uptake to support their own growth and reproduction. The functional profile of CK depicted a typical competitive soil microbiome focused on survival and proliferation. In contrast, pathways such as two-component system signaling, microbial metabolism in diverse environments, antibiotic biosynthesis, secondary metabolite biosynthesis, and carbon metabolism were significantly enriched in the CTG group. This suggested that mycorrhiza formation fostered a metabolically diverse environment, selecting for bacteria with specialized metabolic capacities [53]. Antibiotic production by mycorrhiza-associated bacteria likely protected the symbiotic interface, forming a defensive barrier for the root–fungus consortium [54]. Carbon metabolism enrichment reflected mycorrhiza-induced changes in rhizosphere carbon flow, stimulating bacteria adept at utilizing plant-derived carbon sources [55]. The CTG bacterial community thus exhibited a cooperative and defensive functional profile, characterized by environmental sensing, carbon utilization, and secondary metabolite production to support symbiosis.

Fungal functional prediction using FUNGuild and KEGG pathway analysis (Figure 4B) indicated that pathotroph and saprotroph guilds were enriched in the CK treatment, depicting a micro-environment dominated by resource competition and pathogen pressure [56]. In contrast, the symbiotroph guild was significantly enriched in CTG, confirming successful truffle establishment. As typical ectomycorrhizal fungi, truffles form mutualistic associations that enhance plant access to water and minerals, particularly phosphorus, in exchange for host carbohydrates [6]. *Tuber* colonization not only provided direct symbiotic benefits but also indirectly suppressed pathogens and saprotrophs by modifying the rhizosphere environment and potentially inducing systemic plant resistance. This led to a fungal community shift toward mutualism, establishing a cooperative micro-ecosystem centered on the plant–fungus symbiosis. Successful truffle inoculation reduced pathogen abundance, demonstrating that beneficial symbiotic fungi can enhance plant health through microbiome-mediated biocontrol [22]. Moreover, mycorrhiza improved plant nutrient acquisition, potentially alleviating competition with saprotrophs and reallocating photosynthetic resources toward growth. The CTG fungal community exhibited a specialized and stable functional profile, suggesting that mycorrhiza contributes to a more resilient rhizosphere ecosystem capable of withstanding environmental stresses.

### 2.2. Metabolite Profiling

#### 2.2.1. Global Metabolome Composition

**Figure 5:**
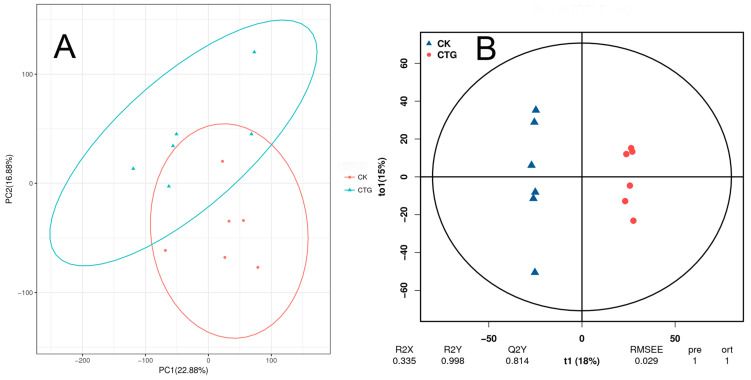
Multivariate analysis of root metabolites from control (CK) and *Tuber*-inoculated (CTG) groups. (**A**) PCA score plot; (**B**) OPLS-DA score plot.

UPLC-MS/MS analysis detected 19,928 metabolic features in root samples from non-inoculated (CK) and *Tuber*-inoculated (CTG) groups. Among these, 3967 metabolites were annotated using the KEGG database and classified into 24 functional categories. The most abundant categories included biosynthesis of other secondary metabolites (445 metabolites), metabolism of terpenoids and polyketides (420), amino acid metabolism (288), and xenobiotics biodegradation and metabolism (245). Principal component analysis (PCA) clearly separated CK and CTG groups along the first and second principal components (PC1: 22.88%; PC2: 16.88% variance). The CK group showed higher within-group variability than CTG (Figure 5A). Orthogonal partial least squares-discriminant analysis (OPLS-DA) further confirmed distinct clustering between groups. The OPLS-DA model exhibited high quality (R^2^Y = 0.998, Q^2^Y = 0.814, RMSEE = 0.029), confirming its robustness and predictive capability (Figure 5B). These results demonstrate pronounced metabolic reprogramming in roots induced by *Tuber* inoculation.

#### 2.2.2. Differential Metabolite Analysis

**Figure 6:**
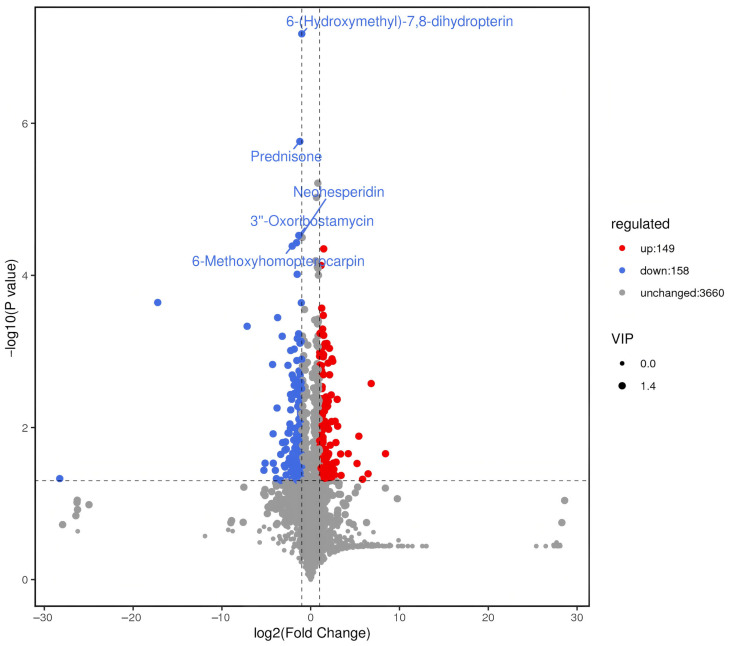
Volcano plot of differential metabolites between control (CK) and *Tuber*-inoculated (CTG) groups.

**Figure 7:**
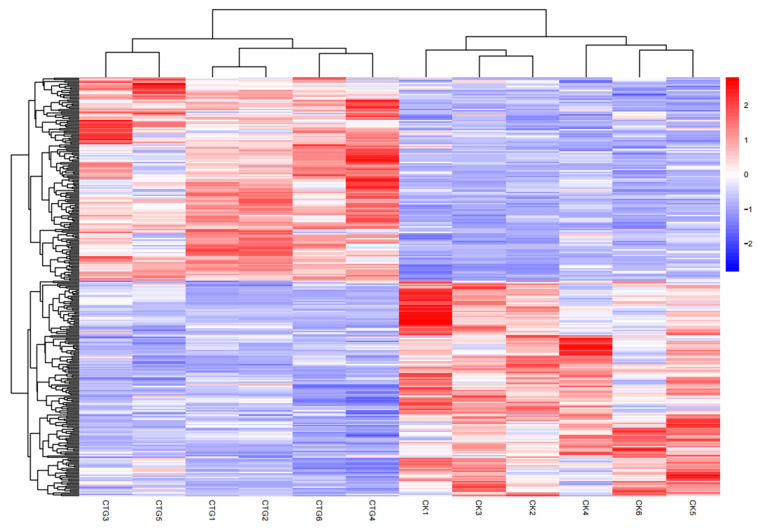
Heatmap of 307 significantly differential metabolites between control (CK) and *Tuber*-inoculated (CTG) treatments.

Differential metabolites between the non-inoculated control (CK) and the *Tuber*-inoculated (CTG) group were identified using thresholds of fold change (FC) > 2, *p* ≤ 0.05, and variable importance in projection (VIP) > 1 from the OPLS-DA model. This stringent approach revealed 307 significantly altered metabolites (149 upregulated, 158 downregulated) out of 3967 annotated compounds (Figure 6; Table S3). Hierarchical clustering heatmap analysis (Figure 7) showed clear separation between CTG and CK, underpinned by distinct metabolic profiles. The upregulation of signaling molecules and phytohormone precursors indicated active molecular dialogue between symbiotic partners [46]. Concurrently, enhanced synthesis of defensive secondary metabolites suggested that the symbiosis actively shapes the microbial community, likely suppressing pathogens and competitors. This offers a biochemical explanation for plant growth inhibition within the “burning zone” (brûlé) of truffle development, implying co-creation of a chemically protected niche by the host and fungus [57]. Furthermore, enrichment of nutrient metabolism intermediates reflected high integration between plant and fungal metabolic networks post-symbiosis. For instance, upregulation of trehalose-6-phosphate, various sugars, and phosphorus-related metabolites indicated efficient nutrient exchange: the fungus aided mineral acquisition (e.g., phosphorus), and the plant supplied photosynthetic carbon [46,58].

**Figure 8:**
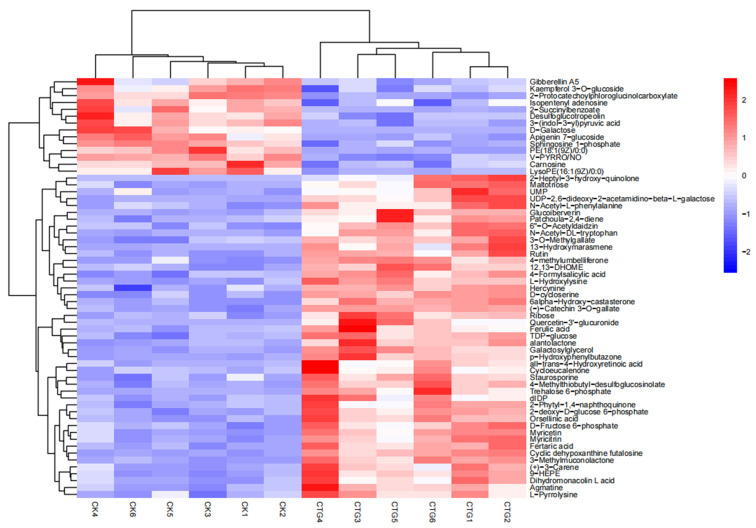
Heatmap of 60 significantly differential metabolites identified in control (CK) and *Tuber*-inoculated (CTG) groups.

A focused hierarchical clustering heatmap (Figure 8) of over 60 differential metabolites involved in hormone signaling, defense, carbon allocation, lipid signaling, and membrane remodeling revealed key reprogramming events:

(1) Hormone Signaling Reprogramming: Shift from “Autotrophic Growth” to “Symbiosis-Priority”.

Gibberellin A5 was downregulated, suggesting resource reallocation away from growth promotion toward the symbiotic interface [59]. In contrast, strigolactone pathway metabolites (e.g., patchoula-2,4-diene, (+)-3-carene) were upregulated, consistent with chemical signaling to promote hyphal branching and symbiosis [60]. Downregulation of isopentenyl adenosine (cytokinin-related) and 3-(indol-3-yl)pyruvic acid (auxin precursor) reflected fine-tuning of the hormonal network for symbiosis [61].

(2) Carbon Reallocation: From “Structural Investment” to “Energy Currency”.

Upregulation of energy currencies like maltotriose, ribose, TDP-glucose, trehalose 6-phosphate, and D-fructose 6-phosphate indicated accumulation of readily utilizable carbon, consistent with fungal provisioning [62,63]. Conversely, downregulation of structural components like D-galactose (a cell wall constituent) suggested reduced cell wall construction, reallocating carbon toward the symbiosis [63,64,65,66].

(3) Defense Strategy: From “Basal Defense” to “Precision Regulation”.

Downregulation of certain flavonoids and phenolics (e.g., kaempferol 3-O-glucoside, apigenin 7-glucoside) indicated suppression of basal defense to facilitate fungal colonization. Upregulation of other phenolics, flavonoids (e.g., rutin, myricetin), and terpenoids (e.g., alantolactone) suggested a shift toward targeted defense, potentially stabilizing the symbiotic interface or providing antioxidant protection.

(4) Lipid Metabolism and Signaling Restructuring.

Downregulation of immune-related signaling lipids—sphingosine 1-phosphate (S1P), lysophospholipids (e.g., LysoPE (16:1 (9Z)/0:0)), and phospholipids (e.g., PE (18:1(9Z)/0:0))—indicated a “signal quiescence” strategy to avoid inappropriate defense against the fungus [65,66].

(5) Cross-Kingdom Signaling and Nutrient Exchange.

Upregulation of the bacterial quorum-sensing signal 2-heptyl-3-hydroxy-quinolone suggested plant perception of microbial community changes, fine-tuning symbiotic strategy. Upregulation of nucleotide synthesis metabolites (e.g., UMP, dIDP) hinted at improved phosphorus and nitrogen availability from the fungus [59,62].

In summary, *Corylus* deployed a coordinated metabolic strategy across hormone signaling, carbon allocation, defense, lipid remodeling, and cross-kingdom communication to establish mutualism with *Tuber*. This involved downregulating autonomous growth and basal defense, reallocating resources toward symbiosis-related signaling (e.g., strigolactones), energy currencies, and specific defenses, while creating an immune-suppressed microenvironment conducive to symbiotic partnership.

### 2.3. Microbiome–Metabolome Correlation Analysis

**Figure 9:**
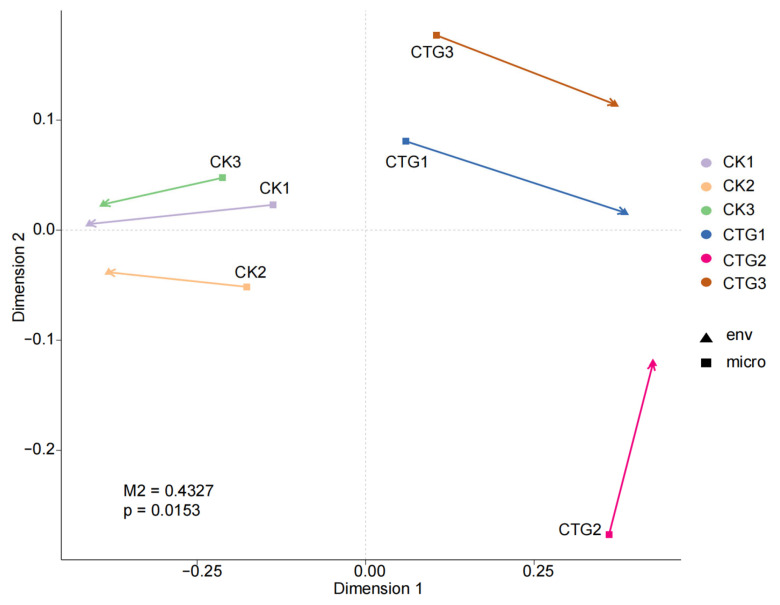
Procrustes analysis reveals congruence between the rhizosphere microbial community and root metabolome in control (CK) and *Tuber*-inoculated (CTG) groups.

Integrated Procrustes analysis revealed a nominal spatial congruence between microbial community structure and metabolome profiles across CTG and CK groups (M^2^ = 0.4327, *p* = 0.0153; Figure 9). Nonetheless, when combined with functional insights from KEGG and FUNGuild analyses, the Procrustes plot provides a visual hypothesis-generating framework for understanding how *Tuber* inoculation may coordinate shifts in both microbial composition and metabolic output. Specifically, bacterial communities shifted from basic metabolic functions toward environmental adaptation and symbiosis support, while fungal trophic modes transitioned from pathotrophy/saprotrophy to symbiotrophy. These changes collectively drove a fundamental restructuring of the rhizosphere metabolome.

The consistency between microbiome and metabolome shifts provides strong evidence that mycorrhization-induced microbial restructuring has tangible functional outcomes—manifested as changes in chemical signaling molecules and metabolic products. This suggests that the plant growth-promotion effect of *Tuber* may not arise solely from direct symbiosis but through a cascading mechanism: the truffle modulates the rhizosphere microbiome into a synergistic, symbiosis-centered community, which in turn supports plant growth via collective metabolic activities such as antibiotic production, signal molecule secretion, and nutrient mobilization. In this context, *Tuber* may act as an ‘ecosystem engineer’ that orchestrates the root microenvironment—not by deterministic control, but by creating conditions favorable for the emergence of a cooperative microbial alliance.

**Figure 10:**
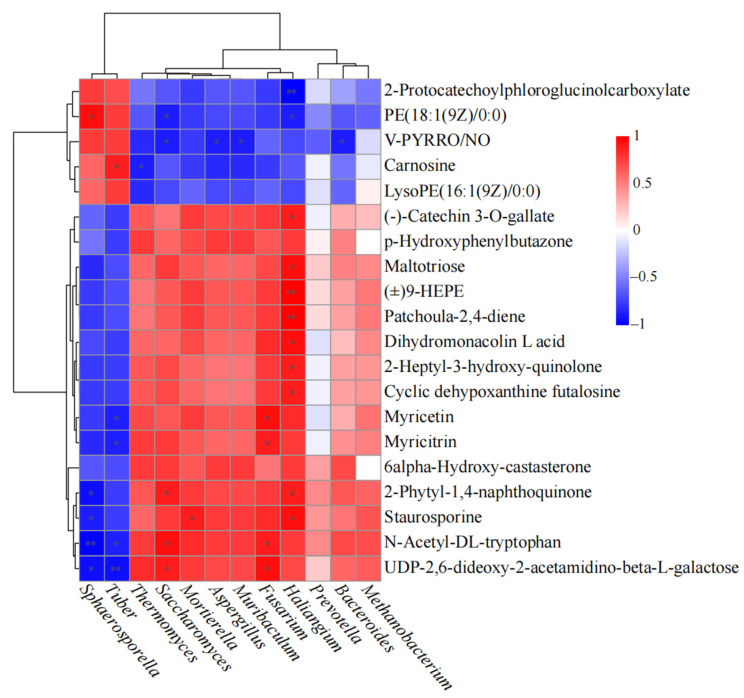
Correlation heatmap between differential metabolites and differential microbial taxa. * indicates *p* < 0.05. ** indicates *p* < 0.01.

Correlation heatmap analysis (Figure 10) visualized interactions between differentially abundant microbial genera and metabolites. Cluster analysis separated microbial taxa into two major branches: fungi and bacteria. Fungi correlated positively with Group A metabolites (e.g., gibberellin A5, kaempferol 3-O-glucoside, D-galactose) and negatively with Group B metabolites (e.g., myricetin, ribose). Bacteria showed the opposite pattern. These distinct correlation profiles reflect a transition in the rhizosphere from a bacterium-dominated competitive environment to a fungus-mediated collaborative system [22], indicating functional differentiation and complementary microbial alliance formation.

Notably, fungi—especially *Tuber* and *Sphaerosporella*—exhibited strong positive correlations with Group A metabolites. *Tuber* was highly significantly correlated (*p* < 0.01) with gibberellin A5 and 3-(indol-3-yl)pyruvic acid (an auxin biosynthesis precursor), suggesting active fungal intervention in host hormonal balance to stimulate root development and expand colonization niches [16]. Positive correlations with carnosine (an antioxidant dipeptide) indicated ROS scavenging to maintain symbiotic integrity [67,68]. Associations with D-galactose and 2-succinylbenzoate (a vitamin K precursor) implied activation of carbon metabolic fluxes supporting structural biosynthesis and energy metabolism [69].

Conversely, fungi correlated negatively with Group B metabolites, which include defense-related compounds like flavonoids and phenolics. Their reduction in mycorrhizal roots reflects successful suppression of basal host defense, fostering an immune-tolerant environment conducive to symbiosis [22]. Significant negative correlations with nucleotide sugars (e.g., TDP-glucose, UDP-2,6-dideoxy-2-acetamidino-beta-L-galactose) and central carbon intermediates (e.g., D-fructose 6-phosphate, trehalose 6-phosphate) indicated substantial carbon diversion toward symbiotic structure formation (e.g., Hartig net, fungal mantle) and fungal metabolic demand [70].

Bacterial genera (e.g., *Thermomyces*, *Mortierella*, *Fusarium*, *Haliangium*, *Prevotella*) clustered separately and correlated negatively with defense compounds (e.g., kaempferol 3-O-glucoside, sphingosine 1-phosphate) and positively with nutrient-exchange molecules (e.g., TDP-glucose, maltotriose) and signaling compounds like 2-heptyl-3-hydroxy-quinolone (a bacterial quorum-sensing molecule). These patterns suggest that truffle mycorrhization drives a functional succession from r-strategist bacteria (dependent on simple plant exudates) toward K-strategists adapted to the fungal-mediated symbiotic environment. The shift in carbon flow from simple root secretions to mycorrhizal-delivered compounds likely reduces bacterial competition for easily degradable carbon, enhancing system stability [36]. Additionally, mycorrhiza-induced suppression of plant immune responses may alleviate antimicrobial pressure on bacteria, facilitating a more cooperative microecological niche [71,72].

The correlation heatmap analysis demonstrates that *Tuber* functions as the core regulator of the *Corylus* rhizosphere micro-ecosystem. The fungal community restructures fundamentally, transitioning from a non-symbiotic state dominated by saprotrophs and pathogens to a symbiotic guild led by *Tuber* and associated mutualistic fungi such as *Sphaerosporella*. This shift indicates a direct and pronounced fungal response to truffle symbiosis. In contrast, bacterial community changes are more subtle: while overall diversity remains high, the functional profile shifts from r-strategists, which rapidly utilize simple resources, toward K-strategists such as Actinobacteriota, which are adept at decomposing complex organic matter. This reflects a functional transition from growth competition to environmental adaptation [73]. The observed disparity suggests that *Tuber* primarily modulates bacterial composition indirectly via metabolic signaling rather than direct suppression, ultimately constructing a more resilient and functionally optimized rhizosphere micro-ecosystem [72].

## 3. Discussion

This study used an integrated microbiome-metabolome approach to profile the multi-level changes induced by *Tuber* inoculation in the *Corylus* rhizosphere micro-ecosystem. Our findings, derived from a controlled pot experiment, illustrate how such a methodological framework can generate testable hypotheses regarding mycorrhiza formation as a process involving not merely a binary plant-fungus symbiosis, but a broader restructuring of the rhizosphere community and its function, potentially orchestrated by the mycorrhizal fungus acting as a “core regulator” [57]. We interpret these findings as a demonstration of the methodology’s utility, while acknowledging that the design limits causal inferences about systemic mechanisms.

### 3.1. Co-Evolution of Microbial Community Structure and Function

A central finding was the “community-specific” effect of mycorrhization on bacterial and fungal communities. The bacterial community maintained alpha-diversity resilience yet underwent significant compositional and functional shifts at both phylum and genus levels [13]. The succession from r-strategist bacteria (e.g., Bacteroidota, Firmicutes) toward K-strategists (e.g., Actinobacteriota) reflected a shift in rhizosphere carbon sources—from simple root exudates to more complex, mycelium-derived compounds [18]. This selectively enriched bacterial taxa capable of decomposing complex organics and adapting to environmental changes.

These results aligned with PICRUSt2 predictions, which showed enrichment in pathways such as “antibiotic biosynthesis” and “secondary metabolism” in the CTG group. This suggested a transition from a bacterial community focused on growth and competition to one that supports symbiotic stability [54]. This “domestication”—rather than disruption—of the bacterial community underscores the indirect regulatory role of mycorrhizal fungi.

In contrast, the fungal community underwent fundamental structural reorganization. Successful *Tuber* colonization established it as the dominant taxon, and through competitive exclusion, significantly reduced fungal diversity and evenness [52]. This “community simplification” did not indicate ecological degradation but functional optimization. The formation of a symbiotic guild centered on *Tuber* and *Sphaerosporella* effectively displaced potential pathogens (e.g., *Fusarium*) and saprotrophs (e.g., *Mortierella*), shifting the dominant fungal trophic mode from saprotrophy/pathotrophy to symbiotrophy [11]—consistent with known mycorrhizal biocontrol effects [22,56]. Together, these structural and functional shifts constructed a more stable and cooperative rhizosphere micro-ecosystem.

### 3.2. Plant Metabolic Reprogramming: The Chemical Basis of Successful Symbiosis

Profound root metabolome changes indicated that mycorrhiza formation involved systemic metabolic reprogramming by the host plant—a core strategy for achieving symbiotic compatibility and efficiency.

First, hormonal fine-tuning was essential. Upregulation of strigolactone metabolites reflected active fungal recruitment [60], whereas downregulation of growth-promoting hormones like gibberellin A5 suggested a shift from autonomous growth to symbiosis priority. Second, strategic carbon reallocation was notable: upregulation of energy currencies (e.g., trehalose-6-phosphate, sugars) and downregulation of structural components (e.g., D-galactose) indicated that the plant redirected carbon from structural investment toward sustaining the mycorrhizal partner [62,63]. Third, defense strategy transformation enabled stable symbiosis: downregulation of basal-defense flavonoids created a colonization-friendly environment, while upregulation of other defensive metabolites (e.g., rutin, gallic acid) may have stabilized the symbiotic interface or provided antioxidant protection [22]. Additionally, downregulation of immune-related lipids such as sphingosine-1-phosphate suggested the plant adopted a “signal quiescence” strategy to avoid inappropriate defense activation against the symbiont [65]. This shift from broad-spectrum defense to precision regulation illustrates the plant’s sophisticated adaptation to symbiotic colonization.

### 3.3. Microbiome–Metabolome Interactions Construct the Symbiotic Microenvironment

Procrustes analysis revealed a significant, moderate-strength correlation between microbial community structure and root metabolite profiles. The correlation heatmap showed that *Tuber*, as the core regulator, correlated significantly with key metabolites (e.g., gibberellin A5, 3-(indol-3-yl)pyruvic acid), suggesting active intervention in host hormonal and metabolic pathways to optimize colonization [16]. Bacterial and fungal communities exhibited opposing correlation patterns with different metabolites, illustrating a post-mycorrhization shift from a “bacterium-dominated competitive environment” to a “fungus-mediated collaborative environment”.

This interaction network implies that the truffle’s growth-promotion effect likely arose not from direct action alone, but through a cascade: *Tuber*, as the pioneer, restructured the rhizosphere microbiome; this reshaped, synergistic “microbial alliance” then indirectly promoted plant growth via collective metabolic activities—such as signaling, antibiotic production, and nutrient mobilization. This offers a deeper perspective on mycorrhizal fungi as “ecosystem engineers” [1].

This study, through an integrated microbiome-metabolome analysis, systematically reveals the multi-level and systemic impact of *Tuber* inoculation on the *Corylus* rhizosphere micro-ecosystem. Our findings strongly suggest that mycorrhiza formation is not merely a simple plant-fungus binary symbiosis, but rather a profound restructuring project of the entire rhizosphere microbial community and its functions, driven by the mycorrhizal fungus acting as a “Core Regulator”.

## 4. Materials and Methods

### 4.1. Experimental Design and Sample Preparation

Yuzhui hazelnut (*Corylus heterophylla* × *C. avellana)* seedlings were propagated from seeds obtained from the Liaoning Province Institute of Economic Forestry. Fresh *Tuber* fruiting bodies were collected from Bijie, China. The method for cultivating mycorrhizal seedlings followed Sillo et al. [74]. Non-inoculated seedlings served as controls (CK). Non-mycorrhizal (CK) and *Tuber*-inoculated mycorrhizal (CTG) were grown under identical greenhouse conditions (a 12-h daily photoperiod with a light intensity of 2000 lux, an average temperature of 23–26 °C, and irrigation with tap water based on soil moisture) for six months without fertilizers or pesticides [75,76]. Rhizosphere soil and lateral roots were collected from six biological replicates per treatment. Soil adhering to the roots after gentle shaking was defined as rhizosphere soil [77]. All samples were flash-frozen in liquid nitrogen and stored at −80 °C.

### 4.2. Microbial Community Analysis

Total DNA was extracted from 0.5 g rhizosphere soil using the SPINeasy DNA Kit (MP Biomedicals, Irvine, CA, USA) following the manufacturer’s protocol. The bacterial 16S rRNA region [78] and fungal ITS1 region [79] were amplified using primer pairs 515F/806R and ITS1-F/ITS1-R, respectively, and sequenced on an Illumina NovaSeq 6000 platform (Illumina, San Diego, CA, USA). Raw reads were quality-filtered with Trimmomatic (v0.39, https://github.com/usadellab/Trimmomatic, accessed on 10 October 2025) [80], merged using FLASH (v1.2.11, http://ccb.jhu.edu/software/FLASH/, accessed on 15 November 2025) [81], and clustered into operational taxonomic units (OTUs) at 97% identity with UPARSE (included in USEARCH v11.0.667, http://www.drive5.com/usearch/, accessed on 20 December 2025) [82]. Taxonomic assignment was performed using the SILVA (bacteria) (v138.2, https://www.arb-silva.de/, accessed on 20 December 2025) [83] and UNITE (fungi) (February 2025, https://unite.ut.ee/, accessed on 20 December 2025) [84,85] databases. Alpha-diversity indices were calculated, and differential taxa were identified via LEfSe analysis [86]. Functional profiles were predicted using PICRUSt2 (version 2.6.3, https://github.com/picrust/picrust2/, accessed on 20 December 2025) for bacteria [87] and FUNGuild (version 1.1, https://github.com/UMNFuN/FUNGuild/, accessed on 20 December 2025) for fungi [88,89].

### 4.3. Metabolomic Profiling

Approximately 50 mg of frozen root tissue (pre-ground to a fine powder in liquid nitrogen) was extracted with 1 mL of cold methanol/acetonitrile/water (2:2:1, *v*/*v*/*v*) containing 0.1% formic acid and a mixture of internal standards. The mixture was sonicated in an ice bath for 10 min, followed by centrifugation at 14,000× *g* for 15 min at 4 °C. The supernatant was collected for subsequent analysis. Separation was performed on a Waters Acquity HSS T3 column (2.1 × 100 mm, 1.8 µm) maintained at 40 °C [27,90]. The mobile phase consisted of water with 0.1% formic acid (A) and acetonitrile (B), using the following gradient program at a flow rate of 0.3 mL min^−1^: 0 min, 100% A → 2 min, 20% A/80% B → 12 min, 100% B → 14 min, 100% B (hold). The injection volume was 5 µL. Mass spectrometry was conducted on a Waters Xevo G2-XS QTOF instrument (Waters Corporation, Milford, MA, USA). Data were acquired in MSE mode, simultaneously collecting data in both positive and negative ion modes [91]. Leucine-enkephalin ([M + H]^+^ = 556.2771, [M − H]^−^ = 554.2615) was used as the lock-spray ion for real-time mass correction, ensuring a mass error of ≤2 ppm. The scan rate was set to 0.2 s for MS and 0.04 s for MS(E), with a collision energy ramp of 20–45 eV. Raw data files were converted to mzML format using ProteoWizard msConvert (version 3.0.25191). Peak detection, alignment (mass tolerance 5 ppm, retention time window 0.2 min), and feature normalization (based on internal standards and total ion current) were performed using Compound Discoverer 3.3. Feature annotation was carried out by matching against the KEGG, HMDB, and METLIN databases (mass tolerance 5 ppm), followed by MS^2^ structural confirmation using MS-Finder (version 1.0.1). The relative peak area was used as a semi-quantitative measure for each metabolite.

### 4.4. Data Integration and Statistical Analysis

Metabolomic data were processed using Progenesis QI (version 2.4) for peak alignment and compound identification against the KEGG database. To ensure the robustness of multivariate analysis with a limited sample size (6 biological replicates per group for metabolomics; 3 replicates for microbiome), the OPLS-DA model was rigorously validated using 200-response permutation tests (RPT); a model was considered valid and not overfitted if the Q^2^ intercept value was below 0.05. Differential metabolites were selected based on a combination of variable importance in projection (VIP) > 1.0 from the validated OPLS-DA model, |fold change| > 2, and *p* < 0.05 from univariate statistical tests [92]. To mitigate the risk of false positives arising from multiple comparisons, *p*-values were adjusted using the Benjamini–Hochberg false discovery rate (FDR) correction. Microbiome–metabolome integration was performed via Procrustes analysis (using the vegan package version 2.6-8 in R) to test overall concordance between the datasets based on Bray–Curtis (microbiome) and Euclidean (metabolome) distance matrices. Subsequently, pairwise Spearman correlations (|ρ| > 0.8, *p* < 0.01) between significantly abundant ASVs and differential metabolites were calculated and visualized in a network to identify robust associations [29]. Statistical significance was evaluated by one-way ANOVA and Duncan’s test (α = 0.05) in SPSS version 26.0. Due to the small N, post hoc comparisons using Duncan’s test are interpreted cautiously and primarily serve to identify consistent directional trends rather than definitive significant differences.

## 5. Conclusions

This integrated multi-omics study demonstrates a workflow for elucidating the ecological mechanisms underlying *Tuber-Corylus* mycorrhizal symbiosis. Mycorrhization exerted a functional impact on the rhizosphere microbial community: while bacterial structure remained stable, its function shifted from r- to K-strategist dominance; the fungal community, however, was fundamentally restructured into a specialized symbiotic guild centered on *Tuber*, which suppressed soil-borne pathogens. The plant adapted through multidimensional metabolic reprogramming—reconfiguring hormone networks, reallocating carbon, transforming defense strategies, and modulating lipid signaling—downregulating autonomous growth and basal defense to prioritize symbiotic signaling, energy supply, and targeted defense, thereby establishing a chemical microenvironment conducive to symbiosis. Despite these advances, three key limitations warrant acknowledgment: (i) the study utilized 6 biological replicates per group for metabolomics and 3 replicates for microbiomics. While this design balances resource allocation with statistical power, the relatively small sample size may limit the generalizability of the findings to diverse ecological contexts; (ii) the 60-day experimental period captures early- to mid-stage symbiotic interactions but does not reflect long-term functional stability under seasonal or climatic fluctuations; (iii) metabolomic detection sensitivity (e.g., low-abundance signaling metabolites) and microbiome sequencing depth (e.g., rare taxa < 0.1% abundance) may have omitted minor but ecologically significant components.

A significant functional linkage emerged between the microbiome and metabolome, with the data supporting a model where *Tuber* acts as a key hub. This framework opens three critical avenues for future research: (i) longitudinal studies spanning 3–5 years are needed to evaluate symbiotic resilience under extreme conditions (e.g., drought, pathogen outbreaks); (ii) expanding metatranscriptomic and spatial metabolomics analyses will clarify transcriptional regulation and spatial metabolite distribution in rhizosphere niches; (iii) replicating findings in heterogeneous field plots (e.g., mixed-age plantations) will enhance translational relevance. From a methodological perspective, this study shows that mycorrhizal symbiosis can be viewed as a synergistic micro-ecosystem, and the approaches used provide a template for generating hypotheses about chemical dialogue between the plant and microbiome. These findings translate into three evidence-based recommendations for forest managers: (i) apply inoculum during seedling establishment to maximize colonization, and use peat-based carriers supplemented with 3% (*w*/*w*) biochar to enhance fungal viability in nutrient-poor soils; (ii) enhance rhizosphere monitoring, deploy IoT-enabled sensors for real-time tracking of soil organic carbon (SOC) and microbial activity, and establish baseline thresholds for pathogen suppression; (iii) apply slow-release phosphorus fertilizers to offset fungal carbon demand while integrating cover crops to enhance rhizosphere nitrogen fixation without competing with host roots. These insights and the applied framework provide a basis for future studies aimed at modulating soil microbiota, enhancing crop stress resistance, and promoting sustainable agriculture via beneficial mycorrhizal introductions. Future studies should explore the functional stability of this symbiosis under varying environmental conditions and its applications in sustainable farming.

## Data Availability

The raw data supporting the conclusions of this article will be made available by the authors on request.

## Supplementary Materials

The following supporting information can be downloaded at: https://www.mdpi.com/article/10.3390/ijms27020768/s1.
